# Supplementary material for: Robust and replicable effects of ageing on resting-state brain electrophysiology measured with MEG
Source: eLife. 2026 Oct 12;14:RP108829. doi: 10.7554/eLife.108829 (PMC13657627; doi:10.7554/eLife.108829)
Supplement: Peer review history

# Editor report: eLife Assessment

**DOI:** 10.7554/eLife.108829.3.sa0

**Authors:** Redmond G O'Connell

**Affiliations:** Trinity College Dublin Ireland

**Keywords:** Compelling, Valuable

The authors ask whether a simple whole-head spectral power analysis of human magnetoencephalography data recorded at rest in a large cohort of adults shows robust effects of age, and their results provide **compelling** evidence that it does. The relative simplicity of the analysis is a major strength of the paper, and the authors are careful to control for many different confounds - although perhaps highly correlated factors like brain anatomy still pose a slight issue. The paper provides a **valuable** power analysis framework that should inform researchers across the broader neuroimaging community.

# Referee report: Reviewer #1 (Public review)

**DOI:** 10.7554/eLife.108829.3.sa1

Summary:

This is a careful, well-powered treatment of age effects in resting-state MEG. Rather than extracting (say) complex connectivity measures, the authors look at the 'simplest possible thing' : changes in the overall power spectrum across age.

Strengths:

They find significant age-related changes at different frequency bands: broadly: attenuation at low-frequency (alpha) and increased beta. These patterns are identified in a large dataset (CamCAN) and then verified in other public data.

Weakness:

Some secondary interpretations (what is "unique" to age vs global anatomy) maybe go beyond what the statistics strictly warrant in the current form, but these can be tightened with (I think pretty quick) additions already foreshadowed by the authors' own analyses.

Aims:

The authors set out to replace piecemeal, band-by-band ageing claims with t-maps, and Cohen's f2 over sensors×frequency ("GLM-Spectrum").

On CamCAN, six spatio-spectral peaks survive relatively strict statistical controls. The larger effects are in low-frequency and upper-alpha/beta ranges (f2 approx. 0.2-0.3), while lower-alpha and gamma reach significance but with small practical impact (f2 < 0.075). A nice finding is that the same qualitative profile appears in three additional independent datasets.

Two analyses are especially interesting. First, the authors show a difference between absolute and relative spectral magnitude (basically within-subject normalization). Relative scaling sharpens spectral specificity of the spatial maps while absolute magnitude is dominated by a broad spatial mode that correlates positively across frequencies, likely reflecting head-position/field-spread factors. The replication of the main age profile is robust to preprocessing decisions (e.g. SSS movement compensation choices) - the bigger determinant of the effect is whether they apply sensor normalization (relative vs absolute).

Second, lots of brain-related things might be related to age and the authors spend some time trying to back out confounds / covariates. This section is handled transparently (in general I found the writing style very clear throughout) - they examine single covariates (sex, BP, GGMV, etc.) and compare simple vs partial age effects. For example, aging is correlated with reductions in global grey-matter volume (GGMV) but it would be nice to find a measure that is independent of this : Controlling for GGMV (via a linear model) reduces age-related effect sizes heterogeneously across space/frequency but does not eliminate them, a nuance the authors treat carefully.

This is a nice paper and I have only a few concrete suggestions:

(1) High-gamma

There can be a lot of EMG / eye movement contamination (I know these were RS eyes closed data but still...) above 30-40 Hz and these effects are the weakest anyway. Could you add an analysis (e.g. ICA/label-based muscle component removal) and show the gamma band's sensitivity to that step. Or just note this point more clearly?

(2) GGMV confound control

Controlling for GGMV reduces, but does not eliminate, age effects. I have a few questions about this: (a) Could we see the residuals as a function of age? I wonder if there are non-linear effects or something else that the regression is not accounting for. Also, (b) GGMV and age are highly colinear - is this an issue? Can regression really split them apart robustly? I think by some cunning orthogonalisation you can compute the effect of age independent of GGVM. I don't think this is the same as the effect 'adjusted' for GGMV (which is what is shown here if I'm reading it correctly). Finally, of course, GGMV might actually be the thing you want to look at (because it might more accurately reflect clinical issues) - so strong correlations are not really a problem: I think really the focus might even be on using MEG to predict GGMV and controlling for age.

Minor presentation edits:

It would be handy to see a single table listing each tested "analysis family" (e.g., sensors×frequency, source parcels×frequency), the multiple control used, and the permutation count. I kept wanting to see this as I was reading to compare back and fore.

I loved the power-planning content (section 3.2, the table with peak f2, CIs, contour plot). I think you could somehow make this even more explicit because people will use it a lot - both for this age/MEG domain and more generally as a template for other types of power planning in the field. Perhaps a "How to use this paper to plan N" guide in a paragraph? Power analysis is surely both "important and difficult" - but also not impossible. A flowchart?

Comments on the latest version:

The authors address all my initial points in their revisions and I have no further comments.

# Referee report: Reviewer #2 (Public review)

**DOI:** 10.7554/eLife.108829.3.sa2

This paper describes application of the "GLM-Spectrum" mass univariate approach to examine the effects of age on M/EEG power spectra. Its strengths include promotion of the unbiased approach, suitable for future meta/mega-analyses, and the provision of effect sizes for powering future studies. These are useful contributions to the literature. What is perhaps lacking is discussion of limitations of this approach, in comparison to other methods.

An analogy is the mass univariate approach to spatial localisation of effects in fMRI/PET images. This approach is unbiased by prior assumptions about the organisation of the brain, but potentially also less sensitive, by ignoring that prior knowledge. For example, a voxelwise univariate approach is less sensitive to detecting effects in functionally homogeneous brain regions, where SNR can be increased by averaging over voxels. In the context of power spectra, the authors' approach deliberately ignores knowledge about the dominant frequency bands / oscillations in human power spectra. This is in contrast to approaches like FOOOF and IRASA, that explicitly parametrise frequency components. I am not saying these methods are better; I just think that the authors should acknowledge that these approaches have advantages over their mass univariate approach (in sensitivity and interpretation; see below). I guess it is a type of bias-sensitivity trade-off: the authors want to avoid bias, but they should acknowledge the corresponding loss of sensitivity, as well as loss of interpretation compared to model-based approaches (i.e., models that parameterise frequency; I don't mean the statistical models for each frequency separately).

An example of the interpretational loss can be seen in the authors' observation of opposite-signed effects of age around the alpha peak. While the authors acknowledge that this pattern can arise from a reduction in alpha frequency with age, this is an indirect inference, and a direct (and likely much more sensitive) approach would be to parametrise and estimate the peak alpha frequency directly for each participant, as done with FOOOF for example (possibly with group priors, as in Medrano et al, 2025, EJN). The authors emphasise the nonlinear effects of age in Fig 2A, but their approach cannot test this directly (e.g. in terms of plotting effects of age on frequency, magnitude, width for each participant), so for me, this figure illustrates a weakness of their approach, not a strength.

Then I think the section "Two dissociable and opposite effects in the alpha range" in the Discussion section is confusing, because if there is a single reduction in alpha peak frequency and magnitude with age, then there is only one "effect", not "two dissociable" ones. If the authors do want to claim that there are two dissociable age effects within the alpha range, then they need to do a statistical test, e.g., that the topographies of low and high alpha are significantly different. This then reveals another limitation of the mass univariate approach - that space (channel) is not parametrised either - so one cannot test for significant channel x effect interactions within this framework, as necessary to really claim a dissociation (e.g., in underlying neural generators).

While the authors show that normalisation of each person's power spectra by the sum across frequencies helps improve some statistics, they might want to say more about disadvantages of this approach, e.g., loss of sensitivity to any effects (e.g. of age) that are broadly distributed across majority of frequencies, loss of real SI units (absolute effect sizes) (as well as problems if normalisation were used for techniques like FOOOF, where the 1/f exponent would be affected).

Please give more information how artifactual ICs were defined. This may be important for cardiac artefacts, since Schmidt et al (2004, eLife) have pointed out how "standard" ICA thresholds can fail to remove all cardiac effects. This is very important for effects of age, given that age affects cardiac dynamics (even though the focus of Schmidt et al is the 1/f exponent, could residual cardiac effects cause artifactual age effects in current results, even above ~1Hz?).

Please could the authors clarify the precise maxfilter arguments, and explain what "reference" was used for the "trans" option - e.g., did the authors consider transforming the data to match a sphere at the centre of the helmet, which might not only remove some of the global power differences due to different head positions, but also be best for generalisation of the effect sizes they report to future studies (assuming the centre of the helmet is the most likely location on average)? And on that matter, did head positions actually differ by age at all?

Comments on the latest version:

I am happy with their revised version.

# Author comment: Author response

**DOI:** 10.7554/eLife.108829.3.sa3

**Authors:** Andrew J Quinn, Jemma Pitt, Oliver Kohl, Chetan Gohil, Mats WJ van Es, Anna C Nobre, Mark W Woolrich

**Affiliations:** University of Birmingham Birmingham United Kingdom; Wellcome Centre for Integrative Neuroimaging Oxford United Kingdom; Institute of Clinical Neuroscience and Medical Psychology, Medical Faculty, Heinrich Heine University Düsseldorf Düsseldorf Germany; Wellcome Centre for Integrative Neuroimaging Oxford United Kingdom; Wellcome Centre for Integrative Neuroimaging Oxford United Kingdom; Yale University New Haven United States; Wellcome Centre for Integrative Neuroimaging Oxford United Kingdom

The following is the authors’ response to the original reviews.

**eLife Assessment**

The authors ask whether a simple whole-head spectral power analysis of human magnetoencephalography data recorded at rest in a large cohort of adults shows robust effects of age, and their results provide compelling evidence that it does. The relative simplicity of the analysis is a major strength of the paper, and the authors are careful to control for many different confounds - although perhaps highly correlated factors like brain anatomy still pose a slight issue. The paper provides a valuable power analysis framework that should inform researchers across the broader neuroimaging community

Many thanks to the reviewers and editorial team. This is an insightful and engaging set of reviews with a range of productive suggestions. We’re pleased that the strengths of this approach show through and that this can be a positive contribution to the community.

We have implemented the large majority of suggestions and believe that the paper is greatly improved with them in place.

**Public Reviews:**

**Reviewer #1 (Public review):**

Summary:

This is a careful, well-powered treatment of age effects in resting-state MEG. Rather than extracting (say) complex connectivity measures, the authors look at the 'simplest possible thing': changes in the overall power spectrum across age.

Strengths:

They find significant age-related changes at different frequency bands: broadly, attenuation at low-frequency (alpha) and increased beta. These patterns are identified in a large dataset (CamCAN) and then verified in other public data.

Weaknesses:

Some secondary interpretations (what is "unique" to age vs global anatomy) may go beyond what the statistics strictly warrant in the current form, but these can be tightened with (I think, fairly quick) additions already foreshadowed by the authors' own analyses.

Aims:

The authors set out to replace piecemeal, band-by-band ageing claims with t-maps, and Cohen's f2 over sensors×frequency ("GLM-Spectrum").

On CamCAN, six spatio-spectral peaks survive relatively strict statistical controls. The larger effects are in low-frequency and upper-alpha/beta ranges (f2 approx 0.2-0.3), while lower-alpha and gamma reach significance but with small practical impact (f2 < 0.075). A nice finding is that the same qualitative profile appears in three additional independent datasets.

Two analyses are especially interesting. First, the authors show a difference between absolute and relative spectral magnitude (basically, within-subject normalization). Relative scaling sharpens the spectral specificity of the spatial maps, while absolute magnitude is dominated by a broad spatial mode that correlates positively across frequencies, likely reflecting head-position/field-spread factors. The replication of the main age profile is robust to preprocessing decisions (e.g., SSS movement compensation choices) - the bigger determinant of the effect is whether they apply sensor normalization (relative vs absolute).

Second, lots of brain-related things might be related to age, and the authors spend some time trying to back out confounds/covariates. This section is handled transparently (in general, I found the writing style very clear throughout) - they examine single covariates (sex, BP, GGMV, etc.) and compare simple vs partial age effects. For example, aging is correlated with reductions in global grey-matter volume (GGMV), but it would be nice to find a measure that is independent of this: controlling for GGMV (via a linear model) reduces age-related effect sizes heterogeneously across space/frequency but does not eliminate them, a nuance the authors treat carefully.

Thank you for this concise summary of the work. We’re glad that the strengths of the approach come through clearly.

This is a nice paper, and I have only a few concrete suggestions:

(1) High-gamma

There can be a lot of EMG / eye movement contamination (I know these were RS eyes closed data, but still..) above 30-40 Hz, and these effects are the weakest anyway. Could you add an analysis (e.g., ICA/label-based muscle component removal) and show the gamma band's sensitivity to that step? Or just note this point more clearly?

Thanks for this suggestion. We agree that there is concern about eye movements for these gamma band analyses. The ICA preprocessing we conducted was relatively thorough, and we were able to remove EOG-related components from the majority of datasets, even though the experimental protocol involved eyes closed at rest. It is possible that some components were missed, but they would require more advanced labelling tools or manual intervention to identify.

There are alternative automated ICA labelling tools that could be used, but these are either not suitable for MEG (ICLabel; https://labeling.ucsd.edu/tutorial, https://mne.tools/mne-icalabel/dev/api/iclabel.html) or optimised for data from CTF/4D systems (MEGNet; https://doi.org/10.1016/j.neuroimage.2021.118402). It is beyond our capacity to modify one of these tools for CamCAN for the current analyses.

It was much more straightforward to rerun the analysis without the ICA step to see the impact of reintroducing all ocular artefacts removed from the v1 analysis. These results are shown in Supplemental section XXX and replicated below.

We have added the following Figure 11 and text to the paper.

Main Text

“We have completed several control analyses to support these findings. Firstly, we have explored the correspondence between alpha peak frequency and the two effects we identified within the canonical alpha range (see supplemental section A.1). Secondly, the overall pattern of findings is consistent in an equivalent source space analysis using LCMV beamforming and parcellation (see supplemental section A.2). Finally, we have repeated the analyses with and without ICA denoising and find that the overall spectral profile is very similar. Effects in low-frequencies, low-alpha and high-gamma are increased with application of ICA, whilst high-alpha and beta remain unchanged and low-gamma effects are reduced (see supplemental section A.3). “

Supplemental materials

“The age results may be contaminated in some way by residual cardiac or ocular artefacts that are not removed during preprocessing. Though ICA denoising was applied, it is possible that some artefactual components were not identified and removed from the dataset. The results at low frequencies and in the gamma range are most likely to be directly impacted by this contamination.

To explore the impact this has on our analysis, we reran the core GLM effect of age on the data with no ICA artefact rejection at all, allowing all eye movements and heart rate components to remain in the data (Figure 10).

This no-ICA analysis has three differences to the original in the publication. The low-frequency decrease with age is stronger and more widespread in the analysis that removes ocular artefacts with ICA. A large negative effect is visible in both analyses, though without ICA several frontal and temporal sensors no longer show significant effects. Similarly, the effect size of the low-frequency effect of age is substantially larger when ICA denoising is applied.

The low-alpha effect was strongly reduced in the analyses that do not remove artefacts with ICA. A large central-occipital group of sensors shows an effect between 7 and 8.5 Hz in the ICA analysis, but this is reduced to a single sensor at 8 Hz when ICA is not computed.

At high frequencies, the age effect in frontal sensors is larger with ICA, and the age effect in posterior sensors is larger without ICA, though the position and frequencies of significant effects are largely unchanged. The remaining effects in the high alpha and beta ranges are unchanged by application of ICA.

Overall, ICA either improves the estimation of age effects (low-frequency, low-alpha, high-gamma) or has negligible effects (high-alpha, beta). Only the posterior low-gamma effect is reduced by ICA. Together, we take this as evidence that our core results are robust to interference by eye movements and that the ICA denoising is working effectively to reduce noise in the analysis.”

(2) GGMV confound control

Controlling for GGMV reduces, but does not eliminate, age effects. I have a few questions about this: (a) Could we see the residuals as a function of age? I wonder if there are non-linear effects or something else that the regression is not accounting for. Also, (b) GGMV and age are highly colinear - is this an issue? Can regression really split them apart robustly? I think by some cunning orthogonalisation, you can compute the effect of age independent of GGVM. I don't think this is the same as the effect 'adjusted' for GGMV (which is what is shown here if I'm reading it correctly). Finally, of course, GGMV might actually be the thing you want to look at (because it might more accurately reflect clinical issues) - so strong correlations are not really a problem: I think really the focus might even be on using MEG to predict GGMV and controlling for age.

This is an interesting area with some tricky interpretation. Thanks for the nudge to help us clarify further.

We have added the following text and Figures 17 & 18 to the paper to clarify these points.

Main Text Section 2.8

“It is important to note that the correlation between age and GGMV does impact the interpretation of the GLM results, but does not prevent the model fit. We explore the model validation and diagnostics in detail in Supplemental Section A.6. In brief, the model is able to separate the unique contributions of age and GGMV. However, the correlation between factors leads to an inflation in the standard error of the estimates. A hypothesis test on these estimates is valid. However, the inflated variance reduces our ability to detect statistically significant partial effects.”

Supplemental Section A.6

“There is a strong correlation between age and Global Grey Matter Volume (Pearson’s r=-0.75). The shared variance arising from this collinearity adds nuance to the interpretation of the results, which we explore in more detail in this section.

Firstly, the sum-square residuals for the group-level model fit including both Age and GGMV are shown as a function of frequency in Figure 17. We see that the residuals broadly follow the overall distribution of variance in the data, peaking at low frequencies and in the alpha range. These frequency ranges are where the strongest signal is visible, but also the highest variability between participants. We would expect that the group model would not perform so well in the points of greatest variability. Importantly, though the residuals are relatively high in the alpha, this is still in the context of a very well-performing model with R2 values of around 80%. “

“Secondly, the correlation between age and GGMV is not inherently problematic for the GLM, but it does add complexity and nuance to the interpretation of the results. Some additional model validation statistics are shown in Figure 18. “

“The singular value spectrum of the design matrix indicates whether a design is low-rank, the smallest singular value in this case in 0.37 which indicates that there isn’t a rank deficiency which would prevent us from estimating the model. Though we can estimate the model, correlated regressors can reduce its efficiency. The variance inflation factors for the joint AGE-GGMV model are above 1 for both parametric regressors, indicating that the standard errors of their estimates are inflated. The VIF of 2.35 indicates that the standard errors of this joint model are around sqrt(2.35) = 1.533 times greater than they would be in a separate or uncorrelated model. Though there is no hard rule for this, the literature generally suggests that a VIF above 5 (or sometimes 10) indicates severe multicollinearity.”

Including additional regressors in the model can change the age estimate by ‘partialling’ out the variance that can be attributed to the other variables and by inflating standard errors. In this specific case of GGMV, the partialled estimates are reduced heterogeneously across space and frequency, and the amount of inflation is at a tolerable level. Overall, the regression is able to separate the unique effects of age and GGMV, at the cost of this inflation in the associated standard errors.”

**Reviewer #2 (Public review):**

This paper describes the application of the "GLM-Spectrum" mass univariate approach to examine the effects of age on M/EEG power spectra. Its strengths include promotion of the unbiased approach, suitable for future meta/mega-analyses, and the provision of effect sizes for powering future studies. These are useful contributions to the literature. What is perhaps lacking is a discussion of the limitations of this approach, in comparison to other methods.

Thank you for this summary and the thoughtful review. The emphasis for this paper is exactly on the points you highlighted, and we’re glad that this has come across well. We agree that a broader comparison to other methods would be a useful addition and have included a series of additional discussion points to address this.

We will take the opportunity to reclarify that our intention for this method is not to replace other, more complex or targeted approaches, but to establish a more generalisable foundation for their development. We’re fully supportive of other approaches and are working on their application ourselves. On reflection, this was not clear enough in the first submission, and we have added the following text to the introduction to clarify.

“Reporting of whole-head and full-frequency spectra of effect estimates would make it straightforward to aggregate across studies and eventually enable identification of sub-threshold effects that may be missed in single analyses but are consistent across studies. We argue that this approach provides a generalisable foundation that can support more complex analyses with a frequency component (such as aperiodic slopes, burst detection, and dynamic functional networks) that require more researcher degrees of freedom.”

An analogy is the mass univariate approach to spatial localisation of effects in fMRI/PET images. This approach is unbiased by prior assumptions about the organisation of the brain, but potentially also less sensitive, by ignoring that prior knowledge. For example, a voxelwise univariate approach is less sensitive to detecting effects in functionally homogeneous brain regions, where SNR can be increased by averaging over voxels.

In the context of power spectra, the authors' approach deliberately ignores knowledge about the dominant frequency bands/oscillations in human power spectra. This is in contrast to approaches like FOOOF and IRASA, which explicitly parametrise frequency components. I am not saying these methods are better; I just think that the authors should acknowledge that these approaches have advantages over their mass univariate approach (in sensitivity and interpretation; see below). I guess it is a type of bias-sensitivity trade-off: the authors want to avoid bias, but they should acknowledge the corresponding loss of sensitivity, as well as loss of interpretation compared to model-based approaches (i.e, models that parameterise frequency; I don't mean the statistical models for each frequency separately).

This is an important point, and we are in complete agreement about the importance of giving a balanced description of how this approach fits within the broader literature. We have added the following paragraph to the discussion on limitations to lay this out more clearly.

“Our approach promotes an exploratory and unbiased approach to quantifying the age effect on neuronal power spectra, which is intended to complement more focused analyses. This has the benefit of reducing researchers’ degrees of freedom and of being broadly generalisable. These come at the cost of a loss in specificity and in sensitivity. Our approach does not specifically quantify features derived from the power spectrum, such as alpha-peak frequency or the aperiodic component of the spectrum. These features are mixed into our full-spectrum estimates but not directly quantified. Thus, they can be challenging to interpret from our approach. Secondly, the mass-univariate approach suffers from a potential loss in sensitivity compared to results that aggregate across spatial or spectral regions that contain consistent results. Where a region or frequency band of interest can be supported from the literature, an approach focusing on a single region has the benefit of reduced noise by averaging estimates from a larger range of observations. Finally, models that consider the whole shape of the spectrum [Donoghue et al. 2021] would also be able to combine information across a range of frequencies rather than depending on a single frequency bin for each estimate. These models have the additional benefit that their parameters are often directly interpretable as features of interest, such as spectral slope or peak frequency.”

An example of the interpretational loss can be seen in the authors' observation of opposite-signed effects of age around the alpha peak. While the authors acknowledge that this pattern can arise from a reduction in alpha frequency with age, this is an indirect inference, and a direct (and likely much more sensitive) approach would be to parametrise and estimate the peak alpha frequency directly for each participant, as done with FOOOF for example (possibly with group priors, as in Medrano et al, 2025, EJN). The authors emphasise the nonlinear effects of age in Figure 2A, but their approach cannot test this directly (e.g., in terms of plotting effects of age on frequency, magnitude, and width for each participant), so for me, this figure illustrates a weakness of their approach, not a strength.

We agree that this point might be misleading within its own figure and have moved the result to the supplemental material with a more lightly phrased wording in the main text. Figure 3 on effect sizes has moved to Figure 2, and a new Figure 3 shows the quadratic effect of age as suggested later in the review.

“We have completed several control analyses to support these findings. Firstly, we have explored the correspondence between alpha peak frequency and the two effects we identified within the canonical alpha range (see supplemental section A.1). Secondly, the overall pattern of findings is consistent in an equivalent source space analysis using LCMV beamforming and parcellation (see supplemental section A.2). Finally, we have repeated the analyses with and without ICA denoising and find that the overall spectral profile is very similar. Effects in low-frequencies, low-alpha and high-gamma are increased with application of ICA, whilst high-alpha and beta remain unchanged, and low-gamma effects are reduced (see supplemental section A.3).“

This supplemental section contains some additional content relevant to the next comment.

Then I think the section "Two dissociable and opposite effects in the alpha range" in the Discussion section is confusing, because if there is a single reduction in alpha peak frequency and magnitude with age, then there is only one "effect", not "two dissociable" ones. If the authors do want to claim that there are two dissociable age effects within the alpha range, then they need to do a statistical test, e.g., that the topographies of low and high alpha are significantly different. This then reveals another limitation of the mass univariate approach - that space (channel) is not parametrised either - so one cannot test for significant channel x effect interactions within this framework, as necessary to really claim a dissociation (e.g., in underlying neural generators).

As above, we agree that this can be misleading and that our intention got muddled in the heading and writing of this subsection. We are not intending to argue that there are definitely two distinct and different effects. We clarify this in the main text of our first version, but not clearly enough:

“The two effects identified in the present analysis may combine to represent a decrease in power and frequency of a single alpha peak”.

We choose to present the first paragraph of this section as a discussion of two effects because this is what is already reported in the literature on changes in alpha power with age. Whilst the decrease in alpha frequency with age is well replicated, the decrease in alpha power is less consistently reported, and the literature shows a highly variable picture of the spatial pattern of this effect. We do not claim a strong dissociation based on these results, but both are reported within the literature.

Our intention is to provide some clarity to this literature by taking a step back and looking at the ‘lay of the land’ in an unbiased way. With this approach, we see different effects of age on magnitude within a canonical alpha range that are completely separated in frequency. With this perspective, it is feasible that different publications taking different regions of interest, frequency band definitions, and processing options could lead to mixed reports of increases and/or decreases in alpha power with age.

When single papers that take focused but inconsistent approaches report inconsistent results, we would argue that our approach leads to a substantial interpretational gain on the level of collections of papers in the literature.

We have added the following content to supplemental section A.1 to clarify and Table 3 illustrates the issue. We have retained the paragraph discussing the possibility that these two effects combine to represent a shift in frequency of a single peak.

Main text section 3.1, replacing the section on ‘Two dissociable effects…’

“Reconciling conflicting reports of the ageing effect on alpha power.

The literature exploring how ageing changes alpha power is heterogeneous. Papers that report results in resting alpha power, either from a canonical band or from an individual peak frequency, include reports of a variety of contrasting age effects. This includes positive correlations with age [Rempe et al., 2023, Stier et al., 2023], negative correlations [Thuwal et al., 2021, Lodder and van Putten, 2011, Medrano et al., 2025, Park et al., 2024], both positive and negative effects separated by space [Hoshi and Shigihara, 2020, Pathak et al., 2022], or null results when correcting for individual frequencies and aperiodic slopes [Scally et al., 2018, Merkin et al., 2023] (see supplemental section A.1 more detailed summary). There is broad variability in methodological approaches, which could account for the variety of results. Stier et al. [2023] suggest that analyses in sensor or source space may lead to different effects. Critically for our work, this variability also prevents formal aggregation of results and meta-analyses that could clarify the picture.

We have proposed that, by taking a step back and tolerating a reduction in sensitivity, we can map out the whole-head whole-frequency structure of the age effect with minimal researcher degrees of freedom and bias. Investigating age effects as a complete spectrum shows that two contrasting age effects on alpha magnitude coexist in close proximity in space and frequency: an increase with a small effect size in central occipital sensors around 7-8.5 Hz and a decrease with a large effect size across a broad set of occipital, temporal and frontal sensors between 9.5-12.5 Hz. Different data samples and different data analysis choices, particularly the selection of regions of interest, source reconstruction, sensor normalisation, or correction of aperiodic components, might emphasise one effect or the other in each analysis. Whilst we have not conclusively explored all possible variants of these analyses, we have provided a framework that would allow future studies to perform formal comparisons and meta-analyses to resolve this bottleneck.”

“Relationship between effects on alpha power and alpha individual frequency.

The two effects identified in the present analysis may combine to represent a decrease in power and frequency of a single alpha peak (Seen qualitatively in Figure 1A). This change in alpha peak frequency is highly replicable [Cesnaite et al., 2023, Dustman et al., 1993, Sahoo et al., 2020, Scally et al., 2018, Pathak et al., 2022, Zibrandtsen and Kjaer, 2021] and is a highly predictive spectral marker of ageing [Stier et al., 2024]. Decreases in alpha peak frequency have been linked to a decline in cognitive performance in healthy ageing [Cesnaite et al., 2023, Finley et al., 2024] and MCI [Garc´es et al., 2013, L´opez-Sanz et al., 2016, Puttaert et al., 2021].

This compelling possibility that the age effect on alpha is a shift in a single peak is complicated by strong evidence for presence of multiple alpha peaks within individuals [Lodder and van Putten, 2011, Chiang et al., 2011, 2008, Klimesch, 1999], with distinct generators and functional relevance [Sokoliuk et al., 2019]. A complex pattern of changes in power, frequency, and spatial distribution likely underlies age-related change in alpha oscillations. Future work will need to explore all three features at the individual level to clearly illuminate the change.”

Supplemental section A.1

“Part of our motivation for this method is that variability in the methodological choices in different publications makes it difficult to aggregate varying results across the literature. For example, though a decrease in alpha peak frequency with increasing age is reported highly consistently, the effect of age on alpha power is much more variable. Table 3 shows a representative sample of publications over the last 20 years that report a change in alpha power with age (note that this is intended to be a representative rather than an exhaustive list). Over half of publications (9/15) report a decrease in alpha power, whilst the remaining publications report an increase (2/15), both increases and decreases (1/15), a decrease but only without correcting for aperiodic slope (1/15), a quadratic effect (1/15), and no effect (1/15). Stier et al. [2023] suggest that the choice of analysis space (sensor space or source reconstruction) is likely a source of discrepancies between studies.

Critically, it is difficult to reconcile these findings with the information reported in the publications. For example, even within the 9 publications that report a decrease, there is little correspondence in the spatial location of the effect. We argue that focused approaches cannot resolve this issue alone, as targeted analyses are more specific to each dataset and less generalisable.

In the specific case of the mixed literature on change in alpha power with age, our results show that both effects are present and separated in frequency. It is feasible that the different methodological choices and datasets used by each study in our survey means that one or other of these two effects were emphasised. As a result, the literature may not be mixed in scientific terms, but that a rich pattern of results is obscured by methodological variability.”

While the authors show that normalisation of each person's power spectra by the sum across frequencies helps improve some statistics, they might want to say more about disadvantages of this approach, e.g., loss of sensitivity to any effects (eg of age) that are broadly distributed across majority of frequencies, loss of real SI units (absolute effect sizes) (as well as problems if normalisation were used for techniques like FOOOF, where the 1/f exponent would be affected).

This is an important point, and we have added the following text to clarify, with one exception. Firstly, the normalisation we applied scales the whole spectrum linearly and would change the 1/f intercept but not the 1/f^alpha exponent.

Main text section 3.3

“Both absolute and relative power measures are used throughout the literature, but there is little consensus about their interpretation [Sandre and Troller-Renfree, 2026]. We focus on relative power for most of our results. By normalising each participant’s power spectrum by the sum across frequencies, we found that the results gained specificity in frequency band and reduced concern about wide intersubject differences in overall variance. Though this improved some analyses, relative power has important drawbacks. In particular, it can reduce sensitivity to effects that are broadly distributed across the spectrum and uses arbitrary scaling rather than meaningful physical units. We support calls in the literature to report both relative and absolute power measures [Rempe et al., 2023, Sandre and Troller-Renfree, 2026].”

The authors should give more information on how artifactual ICs were defined. This may be important for cardiac artefacts, since Schmidt et al (2004, eLife) have pointed out how "standard" ICA thresholds can fail to remove all cardiac effects. This is very important for the effects of age, given that age affects cardiac dynamics (even though the focus of Schmidt et al is the 1/f exponent, could residual cardiac effects cause artifactual age effects in current results, even above ~1Hz?).

Artefactual components were estimated using standard tools in MNE python. Specifically:

https://mne.tools/stable/generated/mne.preprocessing.ICA.html#mne.preprocessing.ICA.find_bads_ecg

https://mne.tools/stable/generated/mne.preprocessing.ICA.html#mne.preprocessing.ICA.find_bads_eog

We have clarified the text in the methods to make the overall process clearer.

We believe that this process has been broadly effective, and we have rejected an average of 2.25 ECG components within each dataset. There remains a strong possibility that residual ECG artefact is present in the data.

We have added the following text to methods section 4.2

“Artefactual components relating to eye movements or the heart rate were automatically identified by correlation with the simultaneous EOG and ECG channels. ECG artefacts were identified using cross-trial phase statistics [Dammers et al., 2008] and an automatic threshold based on the sample rate of the data, as implemented in the mne.preprocessing.ICA.find_bads_ecg function in MNE Python. Between 0 and 3 EOG components were rejected in each dataset, with an average of 0.99 (standard deviation: 0.79) across all datasets. EOG artefacts were identified by correlation with the HEOG and VEOG channels, with a threshold set to r = 0.35, as implemented in the mne.preprocessing.ICA.find_bads_eog function in MNE Python. Between 0 and 5 ECG components were rejected in each dataset, with an average of 2.25 (standard deviation: 0.84) across all datasets. The continuous sensor data were then reconstructed without the influence of the components labelled as artefacts.”

Similar to the response to Reviewer 1, we have not been able to rerun a more advanced ICA algorithm on the data but have repeated the analysis without any ICA to see if including all ocular and cardiac artefacts influences the results. This change does not introduce any new signal components to the results but does attenuate the low-frequency and low-alpha effects. With the assumption that our initial ICA analysis captured the majority of the largest ECG components, we are confident that our core findings are not compromised by cardiac artefacts.

Please see the response to comments to Reviewer 1 for additional text in the manuscript on this point.

The authors should clarify the precise maxfilter arguments, and explain what "reference" was used for the "trans" option - e.g., did the authors consider transforming the data to match a sphere at the centre of the helmet, which might not only remove some of the global power differences due to different head positions, but also be best for generalisation of the effect sizes they report to future studies (assuming the centre of the helmet is the most likely location on average)? And on that matter, did head positions actually differ by age at all?

We have used the maxfilter files as provided by the CamCAN team and an equivalent implementation defined in OSL-ephys for the Oxford and Cambridge MEGUK data. We have clarified the text in section 4.2

“All MEG data pre-processing was carried out using MNE-Python [Gramfort, 2013] and OSL-ephys [Quinn et al., 2022, van Es et al., 2025] using the OSL batch pre-processing tools. For the CamCAN data, we proceeded with analysis on post-maxfilter processed data provided by the CamCAN team. Briefly, the data were processed using AA [Cusack et al. 2015] with automatic bad channel detection (limited to 7 channels) with the origin set to the centre of a sphere fitted to the individual’s Polhemus head shape points. Maxfilter signal-space separation was performed with the temporal extension enabled (temporal window of 10 second and correlation threshold of r = 0.98). Head position was continuously estimated and compensated for during periods where the HPI coils were on. After maxfilter processing, head positions were translated into a default head position defined as a point relative to each individual’s origin in a head coordinate frame. Data with the full maxfilter processing and with the head position translation or movement compensation were extracted from the CamCAN database. An equivalent pipeline was implemented in OSL-Ephys and applied to the data from the MEG-UK datasets from Oxford and Cambridge. Files from the MEG-UK Nottingham dataset were processed with third-order gradiometry applied.”

We found that head position in CamCAN does change with age. We have included the following in Supplemental section A.5

“The head position of participants within the MEG sensor dewar is an important consideration that has the potential to change the signal-to-noise level of each individual data recording. There are significant differences in head position as a function of age in the CamCAN dataset in Y (front-back) direction indicating that older participants are seated further forward in the dewar than younger participants.”

As a point of reference, this pattern is consistent with the largest change with age estimated using the un-normalised raw power spectra in Figure 5. A 1-95Hz region shows a change with age that is consistent with older participants sitting further forward in the dewar. This effect is absent in the relative power contrasts.

We have not fully explored this final point so have not included it in the main paper, but believe it is a useful addition to the discussion of the reviews.

**Recommendations for the authors:**

**Reviewing Editor Comments:**

As you will see, both reviewers are enthusiastic about your paper, indicating that it provides compelling empirical support for its key claims and that it represents a valuable theoretical advance for the field. They provide a number of comments that you may want to consider prior to finalizing the manuscript for publication. All the best, Redmond O'Connell

**Reviewer #1 (Recommendations for the authors):**

(1) It would be handy to see a single table listing each tested "analysis family" (e.g., sensors×frequency, source parcels×frequency), the multiple controls used, and the permutation count. I kept wanting to see this as I was reading to compare back and forth.

This is a helpful suggestion, we have included the table in a new supplemental section which is referenced from the main text at the start of the results

Main text section

“A summary of all GLM analyses carried out in this work is available in supplemental section A.7.”

With the following table included in supplemental section A.7

(2) I loved the power-planning content (section 3.2, the table with peak f2, CIs, contour plot). I think you could somehow make this even more explicit because people will use it a lot - both for this age/MEG domain and more generally as a template for other types of power planning in the field. Perhaps a "How to use this paper to plan N" guide in a paragraph? Power analysis is surely both "important and difficult" - but also not impossible. A flowchart?

We’re very glad that this section is working well and agree that the practical planning content should have been more constructive! We have added the following text as a guide

Main text section 3.2

“We propose the following steps as a practical guide for researchers looking to plan a data sample with a reasonable chance of correctly identifying a particular effect.

(1) Define research question and identify previous results: Your research question must be defined in advance and well specified. There should be relevant data or literature that can be used to guide your decision.

(2) Define the smallest effect size of interest and the decision criterion for the sample decision: It is critical to define the parameters of how you will make your sample size decision ahead of time. We recommend considering what the ’smallest effect size of interest’ [Anvari and Lakens, 2021] would be for your question. This is specific to your question and is about more than statistical significance. What effect size would indicate that there is a practically meaningful effect for the future literature to consider?

(3) Estimate effect sizes to inform your decision: Either by aggregating reported statistics from the literature, or by dedicated processing of previous data, compute an estimate of the effect size. Effect sizes are only estimates, so it is important to compute confidence intervals around your estimate to get a measure of variability.

(4) Compute power/precision curves assuming these results: Using the estimated effect sizes, compute power curves [Baker et al., 2021] that visualise the relationship between effect size, statistical power, and sample size.

(6) Select the sample size that meets your pre-defined criteria: Using your definitions from step 2, and when considering the whole power curve, make a decision about what sample size would give you a reasonable chance of replicating the effect of interest.

(7) Make note of any differences or deviations from this plan during your data collection: There are many practical reasons why your planned sample might not match the data acquired in practice. Such deviations from a plan are ok but should be acknowledged, and any mitigating steps explained [Lakens, 2024].

(8) Document your process for inclusion in a preregistration or publication: Include details on how a sample size decision was made in your research outputs including preregistrations, preprints, and publications. These details will be useful for future researchers to understand your process and to implement their own.”

**Reviewer #2 (Recommendations for the authors):**

(1) Though they mention in the Discussion, the authors could have noted earlier in Section 2.1 that one does not need to assume a linear effect of age - one could use a polynomial expansion or even local splines within the same GLM framework. Indeed, it seems unlikely a priori that effects of age across the wide range of ages in the CamCAN data are linear for all frequencies.

This is an important point, and one that was straightforward to implement in our model. Based on feedback on other sections, we have removed the previous Figure 2, moved Figure 3 on effect sizes to the second position, and added a new Figure 3 containing a model of the quadratic age effects. We believe that this is a substantial improvement in the paper.

Main text section 2.3

“(2.3) Quadratic effects of age

The linear effect of age is a convenient and simple regression model. However, it makes a strong assumption that change with age is uniform across the whole age range. A second group-level GLM was computed with an additional regressor to quantify quadratic effects of age, which have been reported in the ageing literature [G´omez et al., 2013, Rempe et al., 2023, Stier et al., 2023]. The spectrum of t-values for the quadratic age predictor (Figure 3) shows significant effects for a U-shaped change with increasing age in the low frequency (1-5 Hz) range in central sensors. Significant inverted-U shaped effects are present in two frequency ranges in the beta band. A low-frequency beta effect is present in occipital and temporal sensors between 16 Hz and 20 Hz, whilst a second high-beta effect is present in central sensors between 24 Hz and 30.5 Hz. The low-frequency and low-beta effects overlap in space and frequency with linear effects, suggesting that the low-frequency change with age has both a linear increase with age and a U-shaped component, whilst the low-beta effect has a decrease with age and an inverted-U shaped component. The high-beta effect does not overlap in frequency with any of the reported linear effects. The effect sizes for quadratic effects range between Cohen’s F 2 values of 0.033 for low frequency to 0.076 for high beta and are generally lower than the effect sizes for linear change.”

Main text section 2.4

“(2.4) Sample size planning for effects of age on the neuronal power spectrum

We use the 95% confidence intervals around the effect sizes to make recommendations for future sample sizes for future samples that plan to replicate these results. The observed power calculations have no bearing on the interpretation of the present results. Instead, they should be used as a general guideline for planning future studies. Table 1 gives a full summary of the peak statistics and future sample size range for the six age effects identified in Figure 1B. These results have implications for future sample planning for resting-state electrophysiology studies of ageing. Using the upper bound of the sample size estimates as a conservative estimate, the linear changes with age that have relatively large effect sizes would have well-powered replications with sample sizes of around 50-60 participants. However, the smaller linear effects and all the quadratic effects would require samples of 200 or more participants to have the same probability of detecting the effect if it is indeed present (Table 1). This indicates that study samples should be planned with the smallest effect of interest in mind and that ageing effects in different frequency bands may not all be well powered within the same sample.”

Discussion section 3.1

“Linear increase and inverted-U effects in the beta band. The literature consistently reports an increase in low-beta power with age [Gomez et al., 2013, Heinrichs-Graham and Wilson, 2016, Heinrichs-Graham et al., 2018, Hubner et al., 2018, Koyama et al., 1997, Rempe et al., 2023, Stier et al., 2023, Veldhuizen et al., 1993, Xifra-Porxas et al., 2019]. We observed significant inverted-U-shaped quadratic effects of age in two frequency ranges in the beta band, a posterior low-beta component (centred around 18 Hz) and an anterior high-beta component (centred around 25 Hz). This is broadly consistent with reports of quadratic ageing effects in the beta band in the literature [Rempe et al., 2023], though, to our knowledge, our results are the first to suggest a separation of effects into different parts of the beta range. These spectrum changes may be associated with age-related changes in underlying bursting dynamics [Brady et al., 2020, Power et al., 2023].”

“Methods section 4.7

Age plus quadratic age models: To move beyond linear change and explore U and inverted-U shaped effects of age, we fitted a group model with three regressors. One constant term, one z-transformed age regressor, and one z-transformed quadratic age regressor (age − mean(age))^2^.”

(2) The authors could point out an obvious extension of their approach to mixed-effects models, which could properly model within- and between-participant effects (repeated measures), e.g., for longitudinal effects of ageing, GAMMs, etc, including hierarchical linear models that combine trials and participants in the same model, and potentially model trial-specific/stimulus effects, etc.

This is an important point; we have added the following paragraph to the discussion section.

Discussion section 3.5

“Future extensions

A clear future extension for this work is to formally incorporate estimates of within-subject variability into a mixed-effect model. These powerful models would enable modelling of both fixed effects and random effects, allowing researchers to account for variation within individuals over time and between individuals. LMMs also provide improved approaches for handling missing observations and unbalanced designs, making them especially useful for longitudinal and hierarchical data. A second expansion of this work could use Generalised Additive Mixed Models (GAMMs) to model non-linear relationships using smooth functions while also accounting for random effects. This may allow for more realistic representations of complex patterns of change across age. Linear Mixed Modelling comes with a substantial increase in researcher degrees of freedom and can be challenging to implement and report accurately [Meteyard and Davies, 2020]. We have used fixed-effects modelling in this work in line with our objectives of maintaining generalisability and minimising researcher degrees of freedom.”

(3) Do the authors want to comment on why effect sizes in Figure 4Ci are so much higher for the Oxford sample? I know the authors' main point is that smaller samples lead to more variable estimates of the true effect size, but there are also other interpretations for these sample differences, e.g., recruitment differences, scanner differences, etc. Have the authors considered implementing empirically Bayesian approaches (like COMBAT, a type of mixed-effects model) to adjust for site differences in both offset and scaling, which I think should be a fairly simple extension of GLM-spectrum?

We agree that our explanation of here is somewhat lacking. To be transparent, we have thought long and hard about this difference and cannot identify a clear reason why this difference is so striking. There is no apparent difference in the other covariates, such as head position, age, or gender, which could explain why the Oxford dataset has larger effect sizes in this specific frequency range (the results in the low-frequency and alpha ranges are consistent).

We have added the following to the main text to highlight dedicated data harmonisation strategies that could be applied in this situation.

Main text section 2.5

“This may arise from relatively poor estimates of the population level variability from smaller data samples. It is possible that more systematic differences in the participant sampling, recruitment, and data acquisition process could contribute to between-site differences. At present, our analyses can show that the core effects of ageing are replicable across several datasets, though we have not formally combined these datasets into a single analysis. Formal methods for data harmonisation, such as ComBat [Johnson et al., 2006], could correct for additive and multiplicative differences in data scaling across sites to improve site comparisons.

(4) There are a number of formatting problems - at least in the PDF provided to reviewers - for some symbols, e.g., "below ¡7 Hz" (I presume "j" was originally "~" or something?). Also, sometimes a figure is cited by a single, hyperlinked number, without the word "Figure".

(5) Line 402: "influence" = "influenced"

(6) Line 431: date for Gelman & Loken?

Thank you for highlighting these, we have done a thorough proofread and fixed a large number of spelling and formatting issues.
